# The current state, challenges, and future directions of artificial intelligence in healthcare in Saudi Arabia: systematic review

**DOI:** 10.3389/frai.2025.1518440

**Published:** 2025-04-07

**Authors:** Najla M. Aljehani, Fatima E. Al Nawees

**Affiliations:** ^1^Department of Public Health, College of Health Sciences, Saudi Electronic University, Riyadh, Saudi Arabia; ^2^Department of Public Health, College of Health Sciences, Saudi Electronic University, Dammam, Saudi Arabia; ^3^Department of Respiratory Therapy, Mohammed Al-Mana College for Medical Sciences, Dammam, Saudi Arabia

**Keywords:** artificial intelligence in healthcare, future of artificial intelligence, state of artificial intelligence, artificial intelligence technology, artificial intelligence

## Abstract

**Background:**

The use of artificial intelligence has been part of the healthcare technologies used in managing various aspects of healthcare processes. In Saudi Arabia, the use of artificial intelligence for managing healthcare has been influenced by the increasing use of healthcare technologies within the healthcare system. The aim of this study is to systematically review the current state, challenges, and future directions of artificial intelligence in healthcare in Saudi Arabia.

**Methods:**

The study used a systematic review methodology, which used the critical appraisal of articles on the use of artificial intelligence in healthcare. The critical appraisal used the Preferred Reporting Items for Systematic Reviews and Meta-Analyses (PRISMA) and Joanna Briggs Institute (JBI) to implement the inclusion and exclusion criteria. The initial search for articles led to 88 articles, which were screened to 13, based on the inclusion and exclusion criteria.

**Results:**

The current state of the use of artificial intelligence in Saudi’s healthcare system has been slowed down by the gradual uptake of healthcare technologies and the investments required. The main challenges identified included lack of policies to support artificial intelligence, lack of adequate capital for infrastructure and human resources and lack of cultures to accommodate the artificial intelligence in Saudi Arabia. With the current privatization and increased use of the artificial intelligence, the future of artificial intelligence in Saudi’s healthcare system would see an increase in their utilization. Specific findings indicate the potential of artificial intelligence in improving clinical practice through blockchain, and that investments in artificial intelligence have encompasses various applications, including radiology. Skills gaps expected among healthcare professionals and the adoption of new technology are difficulties impacting the utilization of artificial intelligence in the healthcare sector.

**Conclusion:**

The use of artificial intelligence in Saudi’s healthcare system requires the investments into infrastructure, human resource development and gradual commitments towards the healthcare technologies. The use of artificial intelligence would have benefits such as effectiveness in access to care and ability to meet the healthcare outcomes.

## 1 Introduction

The term “artificial intelligence” is broad and lacks a universally accepted definition. Artificial Intelligence (AI) can be defined as a computational system that exhibits the ability to effectively address intricate problems or execute actions in order to achieve predetermined objectives within particular contexts. In alternative terms, artificial intelligence encompasses a comprehensive array of tools, methodologies, and approaches that aim to endow computers with human-like capabilities. AI encompasses various areas, including machine learning (ML), deep learning, natural language processing (NLP), and rule-based systems, each offering unique approaches to problem-solving (Chikhaoui et al., 2022).

Artificial intelligence is gaining attention in medicine, with Clinical Decision Support Systems (CDSS) being developed since the mid-20th century. CDSS are computer-based systems designed to assist clinicians in making diagnostic and treatment decisions. It is intended to enhance healthcare delivery by augmenting medical judgments with specific clinical information (Aboalshamat et al., 2022). Rule-based techniques have been effective in interpreting electrocardiogram (ECGs), disease diagnosis, and treatment selection. Recent studies have used machine learning and deep learning for pattern recognition and image identification, largely attributed to advancements in deep learning techniques (Aboalshamat et al., 2022). In addition, the integration of AI is significantly impacting the healthcare sector with methodologies such as machine learning algorithms, deep learning approaches, and processing of natural language strategies (Alotaibi and Alshehri, 2023). Currently, there is a growing body of evidence suggesting that AI provides significant potential in the fields of medical diagnosis, prognosis prediction, and the development of personalized treatment plans for patients (Secinaro et al., 2021). The successful integration of these technologies’ hinges substantially on patient perspectives and acceptance, necessitating robust evaluation protocols and strategic implementation frameworks (Amer et al., 2022a).

The use of artificial intelligence in Saudi Arabia has been increasing, due to the need for technological advancements in the country. Saudi Arabia’s Vision 2030 is a strategic framework that was developed in 2016, designed to reduce Saudi Arabia’s dependence on oil, diversify its economy, and develop public service sectors such as health, education, infrastructure, recreation, and tourism (Alasiri and Mohammed, 2022). The application has increased in the healthcare systems that capitalize on additional intelligence to improve effectiveness. According to the research on the factors that determine the use of AI in the healthcare systems, the focus in Saudi Arabia has been on the need for investments to assist actualize its utilization (Khafaji et al., 2022). In Saudi Arabia, the emphasis in the healthcare technologies has been integral in promoting the mandates and roles of AI in the healthcare system. The aim would be to generate practices that can deliver the intended goals, including in the gradual infrastructure investments (Khafaji et al., 2022). E-learning experiences during COVID-19 pandemic offer valuable frameworks for conceptualizing AI integration in healthcare education systems (Mahamid et al., 2022). Moreover, AI systems have the capability to employ advanced algorithms in order to acquire knowledge from extensive healthcare datasets. This enables them to offer current medical information, minimize errors in diagnosis and treatment, and extract valuable insights from a substantial patient population. While AI systems can aid in making timely inferences regarding health risk alerts and predictions of health outcomes (Secinaro et al., 2021), the active participation of healthcare workers in the evaluation process remains crucial, as their clinical insights directly inform effective integration strategies for these AI technologies (Amer et al., 2022b).

In a survey study on the challenges that come with the use of AI in Saudi Arabia, the healthcare systems have been identified as having gaps in accommodating technologies. The challenges would come from lack of adequate approaches for integrating AI into the healthcare systems (Al-Jehani et al., 2021). The system has been investing in promoting accessibility, which prioritizes costs and access to healthcare. The gaps in prioritizing technologies have led to challenges in meeting the expected AI interventions and investments (Nasseef et al., 2022). In addition, the negative factors that have affected the use of AI in Saudi Arabia include lack of culture for using AI, poor investments in AI, and lack of adequate structures to accommodate the AI (Rong et al., 2020). The development of the AI would focus on the structural components that can help meet the needs and goals that relate to the healthcare implementation (Rong et al., 2020). Furthermore, it is important to recognize the broader context of the Fourth Industrial Revolution and the role of intellectual capital in driving the successful adoption of AI. The development of the intellectual capital is part of the factors that determine the use of AI in Saudi Arabia (Al Momani et al., 2021).

The future of AI focuses on the current increase in the use of technologies in managing healthcare processes. Countries including Saudi Arabia focuses on the use of technologies as part of implementing the vision 2030. While this strategic framework provides crucial insights into technological integration for healthcare advancement (Abdullah and Fakieh, 2020), its success fundamentally depends on understanding the complex interplay between AI, intellectual capital, and the evolving landscape of business, education, and healthcare systems (Al Momani et al., 2021).

Despite the increasing adoption of AI, there remains a lack of systematic reviews specifically examining its application within Saudi Arabia’s healthcare system. While existing literature introduces AI concepts and outlines the prerequisites for implementation, there is a noticeable gap concerning detailed analyses of key AI applications and challenges. Limited attention has been given to the intersection of AI, the Internet of Things (IoT), and the healthcare sector, particularly regarding mental health and the future of education within this evolving landscape (Saleem et al., 2023). However, the effective application of AI requires a harmonious combination of technological tools, human capital and collaboration for effective use and governance (Nour et al., 2022; Saleem et al., 2023).

### 1.1 Research aim

The aim of this study is to systematically review the current state, challenges, and future directions of artificial intelligence in healthcare in Saudi Arabia.

### 1.2 Research questions

- What is the current utilization of artificial intelligence in Saudi Arabia’s healthcare system?
- What are the current challenges facing the use of artificial intelligence in Saudi Arabia’s healthcare system?
- How is the future direction that would guide the use of artificial intelligence in Saud Arabia’s healthcare system?

### 1.3 Research objectives

The objectives that guide the research are as follows:

- To determine the current utilization of artificial intelligence in Saudi Arabia’s healthcare system.
- To assess the current challenges encountering the use of artificial intelligence in Saudi Arabia’s healthcare system.
- To determine the future direction that would guide the use of artificial intelligence (AI) in Saud Arabia’s healthcare system.

## 2 Study method

### 2.1 Study design

The design for the current research is a systematic review, which comes from the use of the medical literature to assess the use, current challenges and future directions for the use of AI in healthcare systems in Saudi Arabia. The review will appraise the medical literature that provides information on the identified variables in the research topic.

### 2.2 Search strategy and databases

The current systematic review targets the development of comprehensive search for the articles in databases such as Saudi Journal of Medicine, PubMed, Elsevier, Scopus, Taylor and Francis, Journal of Family Medicine and Embase. The key words used in the search include Use of AI in Saudi’s Healthcare, Challenges of AI in Saudi’s Healthcare, Opportunities for AI in healthcare and the future of AI in Saudi Arabia. The study selection focuses on articles with defined methods of data collection, populations and findings relating to the utilization, challenges, and future directions of AI in healthcare. The application of the PRISMA flow diagram helped in selecting the studies that met the criteria developed in the inclusion and exclusion criteria. The scope of the research is guided by the healthcare system in Saudi Arabia and the applications of AI in the healthcare system.

### 2.3 Inclusion criteria

The inclusion criteria are as follows: publications and articles that provide information on the utilization of AI in Saudi’s public and private healthcare system. Articles on the healthcare settings such as clinics and hospsitals. Articles on the challenges and future directions of AI in the healthcare sector. Articles published between 2018 and 2023. Qualitaitive and quantitative studies with defined methods of data collection.

### 2.4 Exclusion criteria

The exclusion criteria for the research include the following: publications and articles that do not include information that relates to the use, challenges, and the future of AI in Saudi healthcare system. Review articles and systematic review studies. Studies with significant bias and methodological flow. Studies with incomplete findings. Publications and articles published older than 2018. Articles not written in English language.

### 2.5 Ethical consideration

In this systematic review, the only consideration to consider and use is the bias, which comes from the approaches used when utilizing medical literature. The bias could come from the selection process, which can affect objectivity in the research. The use of critical appraisal tools and the research objectives would help in reducing the occurrence of bias.

### 2.6 Quality assessment

The Joanna Briggs Institute (JBI) critical appraisal checklist was used to minimize bias and assess the methodological rigor of each of the 13 included studies (Munn et al., 2020). The JBI checklist consists of eight critical appraisal questions answered with yes or no. The result of the checklist is shown in Table 1, which determines the ability for each of the articles identified form the PRISMA flow diagram.

**Table 1:** JBI assessment.

Authors	Q1	Q2	Q3	Q4	Q5	Q6	Q7	Q8	Score
Abdelmaboud et al. (2022)	Y	Y	Y	Y	Y	Y	Y	N	87.5%
Abdullah and Fakieh (2020)	Y	Y	Y	Y	Y	Y	Y	Y	100%
Aboalshamat et al. (2022)	U	Y	Y	Y	Y	Y	Y	Y	87.5%
Alharthi (2018)	Y	Y	Y	Y	Y	Y	Y	Y	100%
Al-Jehani et al. (2021)	Y	Y	Y	Y	Y	U	Y	Y	87.5%
Alotaibi and Alshehri (2023)	Y	Y	Y	Y	Y	Y	Y	Y	100%
Alowais et al. (2023)	Y	Y	Y	Y	Y	Y	Y	Y	100%
Chikhaoui et al. (2022)	Y	N	Y	Y	Y	N	Y	Y	75%
El-Sherif et al. (2022)	Y	Y	Y	U	Y	Y	Y	Y	87.5%
Khafaji et al. (2022)	N/A	Y	Y	Y	Y	Y	Y	Y	87.5%
Nasseef et al. (2022)	Y	Y	Y	Y	Y	Y	Y	Y	100%
Pugliese et al. (2021)	Y	Y	Y	Y	Y	Y	Y	Y	100%
Rong et al. (2020)	Y	Y	Y	Y	Y	U	N	Y	75%
Salah et al. (2019)	N	Y	Y	Y	Y	Y	Y	Y	87.5%

### 2.7 Data extraction

**Figure 1:**
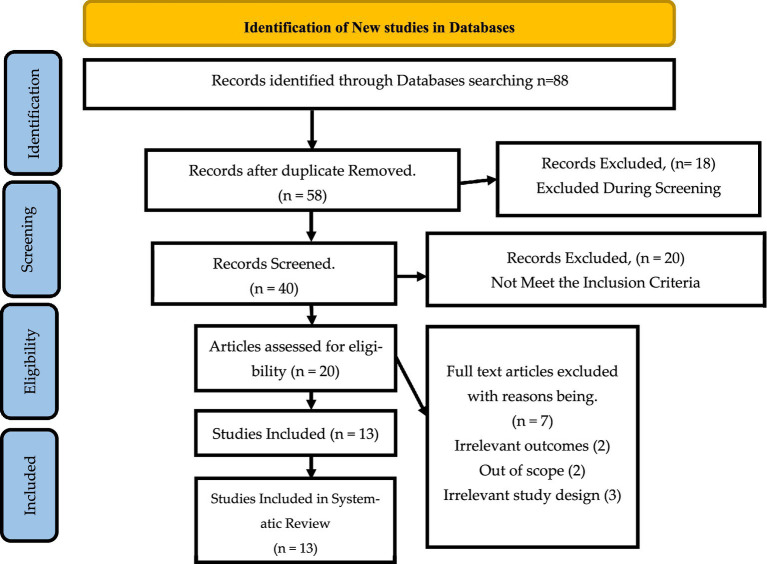
PRISMA flow diagram for identification of relevant studies.

The data extraction process for this systematic review was structured according to the PRISMA flow diagram shown in Figure 1 in alignment with the research questions. The PRISMA diagram outlines the methodology used to identify and select relevant studies based on established inclusion and exclusion criteria (Abdullah and Fakieh, 2020). These criteria were crucial in determining the specific data to be extracted from each of the 13 studies included in the review.

For instance, the inclusion criterion requiring studies to focus on the use of AI in Saudi Arabia’s healthcare system influenced the extraction of data concerning the specific AI applications discussed in each article. Likewise, the criterion addressing challenges and future directions shaped the extraction of data related to barriers to AI adoption and the prospects for future development.

The extracted data from the selected studies, in line with the PRISMA diagram, covered key aspects such as the utilization of AI in the Saudi healthcare system, existing challenges, and potential future directions. These themes were carefully assessed to identify and illustrate the relevant variables. The data was all summarized to provide a comprehensive review.

### 2.8 Data analysis

Data analysis considers the thematic analysis, whereby the articles will be evaluated to determine the themes and their alignment to the current research. The data will be organized based on the themes, which can be related to each of the objectives identified. The data analysis for this systematic review considers the application of the PRISMA and JBI checklist. The PRISM flowchart will help with screening the articles based on duplications and eligibility. The JBI checklist will help to assess the articles based on the checklist questions to determine their qualifications in the current review. The data analysis will use the research objectives that would guide the determination and discussion of the findings developed in the research.

## 3 Results

### 3.1 PRISMA flow diagram

The search for the relevant data to include started with 88 articles, which were collected from online databases that were identified above. A total of 30 articles were eliminated due to duplication, while 18 articles were excluded during the screening. Only 20 articles met the inclusion criteria, with 7 articles ben eliminated for irrelevant study design, irrelevant outcomes and being out of context. The articles included in the review were 13. Figure 1 shows the PRISMA flow diagram for identification of relevant studies.

### 3.2 JBI assessment

The Joanna Briggs Institute (JBI) assessment is important in the evaluation of the qualitative aspects of the articles developed and used in the review. The JBI checklist consists of eight questions that help in assessing the quality of the articles selected for use in the research. Table 1 shows the JBI assessment of the 13 articles identified form the PRISMA flow diagram.

### 3.3 General characterization table

The characterization of the various studies has focused on the different perspectives that can inform on the current state, future and challenges of the use of AI in the healthcare sector (Table 2). These studies were published between 2018 and 2023, with a significant concentration of publications between 2022 and 2023 as shown in Table 3. The majority of studies (9 out of 13) were published in this period, demonstrating the rapid research in AI applications within Saudi Arabian healthcare. The use of blockchain technology has developed the baselines for incorporating AI, through the data management systems (Abdelmaboud et al., 2022). The use of AI was related to the healthcare revolution strategies used for the management of clinical practice (Alowais et al., 2023). The implementation process has to consider the legal and ethical implications, given their role in determining compliances (Chikhaoui et al., 2022). During the COVID-19 pandemic, the use of AI helped to incorporate the roles and mandates of the telehealth systems to help promote access to health interventions, including in emergencies (El-Sherif et al., 2022). The investments in AI have incorporated different uses, such as radiology and in managing the specific healthcare systems. Such approaches have influenced the management and applications involved in addressing healthcare needs (Khafaji et al., 2022; Nasseef et al., 2022).

**Table 2:** The characterization of the articles included for the systematic review.

Authors	Title	Study type	Sample	Main findings
Abdelmaboud et al. (2022)	Blockchain for IoT applications: taxonomy, platforms, recent advances, challenges and future research directions.	Cross Sectional	27 participants in private facilities in Saudi Arabia.	The increasing use of IoT has led to the contextualization of AI based on sectorial approaches.
Abdullah and Fakieh (2020)	Health Care Employees’ Perceptions of the Use of Artificial Intelligence Applications.	Cross Sectional	100 employees in public hospitals in Riyadh, Saudi Arabia.	The healthcare employees indicate positive perceptions in the use of the AI in improving healthcare services
Aboalshamat et al. (2022)	Medical and Dental Professionals Readiness for Artificial Intelligence for Saudi Arabia Vision 2030.	Cross Sectional	200 employees from public and private healthcare sectors in Makkah, Saudi Arabia.	The study indicates that most medical professionals lacked the preparedness for integrating and working with the AI as part of developing healthcare strategy.
Alharthi (2018)	Healthcare predictive analytics: An overview with a focus on Saudi Arabia.	Experimental	300 participants from healthcare workers from public healthcare system in Saudi Arabia.	The development of predictive analytics enables the realization of the intended goals and roles in actualizing healthcare development.
Alotaibi and Alshehri (2023)	Prospers and Obstacles in Using Artificial Intelligence in Saudi Arabia Higher Education Institutions—The Potential of AI-Based Learning Outcomes.	Cross Sectional	15 institutions in Saudi Arabia.	The prospers have included increased investments, with the cultures and expectations from the patients creating the main obstacles.
Alowais et al. (2023)	Revolutionizing healthcare: the role of artificial intelligence in clinical practice.	Cross Sectional	220 participants from public healthcare in Saudi	The role of AI in clinical practices included diagnoses, interventions and developing follow ups.
Chikhaoui et al. (2022)	Artificial Intelligence Applications in Healthcare Sector: Ethical and Legal Challenges.	Cross Sectional	50 participants from private hospitals in Saudi	The ethical and legal challenges affect the commitments towards integrating AI, due to risks and vulnerabilities that affect the quality of the outcomes.
El-Sherif et al. (2022)	Telehealth and Artificial Intelligence insights into healthcare during the COVID-19 pandemic.	Cross Sectional	80 participants from public hospitals.	The COVID-19 pandemic has been instrumental in promoting the appreciation of the role of AI in promoting access to care.
Khafaji et al. (2022)	Artificial intelligence in radiology.	Cross Sectional	20 radiologists from private health facilities in Saudi Arabia.	The use of AI in radiology has enabled an improvement in the overall quality of care.
Nasseef et al. (2022)	Artificial intelligence-based public healthcare systems: G2G knowledge-based exchange to enhance the decision-making process.	Cross sectional	40 participants from public health facilities in Saudi Arabia.	Healthcare organizations have invested in the development of strategic criteria for developing healthcare through AI.
Pugliese et al. (2021)	Machine learning-based approach: Global trends, research directions, and regulatory standpoints	Quasi Experiment	18 participants from private hospitals.	The development and use of machine learning can help actualize the demands in healthcare.
Rong et al. (2020)	Artificial intelligence in healthcare: review and prediction case studies.	Case Study	11 case studies from Saudi’s healthcare system	The use of AI would increase in healthcare development due to the commitments made towards sustaining technological use.
Salah et al. (2019)	Machine learning applications in the diagnosis of leukemia: Current trends and future directions.	Cross Sectional	90 participants from private hospitals.	The concepts of machine learning can help in improving healthcare.

**Table 3:** Publication year distribution of included studies.

Publication Year	Number of Studies
2018	1
2019	0
2020	2
2021	2
2022	6
2023	2
Total	13

The role of healthcare professionals has also been identified as part of the influences of the current and future use of AI in healthcare (Abdullah and Fakieh, 2020). The gaps in skills expected and the acceptance of new technologies are some of the challenges affecting the use of AI by healthcare professionals (Aboalshamat et al., 2022). The role of the institutions of higher learning has been integral in determining the opportunities for realizing the intended AI applications (Alotaibi and Alshehri, 2023).

For healthcare systems, the development of technological systems such as healthcare predictive would determine the factors that would determine the subsequent development of AI (Alharthi, 2018). The adoption of machine learning has been recognized as an influence on the realization of the AI implementation (Pugliese et al., 2021). The application of machine learning processes has remained effective in addressing the requirements for promoting an effective strategy to accommodate the intelligence inputs in healthcare (Salah et al., 2019). The predictions on the use of AI in healthcare indicates the increased use, based on the increasing use of healthcare technologies in the healthcare sector (Rong et al., 2020).

## 4 Discussion

From the results, the development and use of artificial intelligence came with different concepts and procedures that would influence the actualization of the healthcare delivery processes. The creation of the policies and measures that would influence the actualization of the healthcare processes determines the approaches and strategies to influence the healthcare quality. The current utilizations have focused on the need to address the healthcare quality needs in the system. The current and future challenges were identified based on the existing measures for improving the use of AI in the Saudi healthcare system.

### 4.1 What is the current utilization of artificial intelligence in Saudi Arabia’s healthcare system?

The results showed that the use of the healthcare technologies has been increasing in Saudi Arabia, focusing on the investments toward contemporary technologies (Khafaji et al., 2022). These technologies have included Artificial intelligence, which has been incorporated in the information systems within the healthcare systems. The focus on investments has been influenced by the demand to integrate the various technologies in managing healthcare. These findings align with previous research indicating that successful AI implementation requires a robust technological ecosystem and a skilled workforce (Khafaji et al., 2022; Saleem et al., 2023). During the COVID-19 pandemic, the development of artificial intelligence increases, due to the ability to promote remote systems and the creation of effective measures for improving access to the healthcare services (Khafaji et al., 2022). The pandemic created the need for consistent investments in strategies and measures that would lead to the development of the required healthcare services for the population.

In addition, the findings showed that the use of the AI in Saudi’s healthcare system has focused on the different procedures for managing the healthcare processes. The inclusion of telemedicine and remote monitoring strategies have come with the need to incorporate the AI technologies (El-Sherif et al., 2022). The technologies have led to the ability to build connectivity between the patients and the hospitals, which has been an important factor when establishing patient monitoring. The intended benefits from AI would include the ability to develop and influence healthcare management. The significance of the AI in such cases would be to encourage the commitments that come with the realization of the intended benefits from the AI (El-Sherif et al., 2022). The hospital processes capitalize in the technologies to help promote efficiency and effectiveness, which can be derived from the use of the AI systems. The investments in AI have also led to the advancements in the research factors that would influence the realization of sustainable development in the healthcare systems.

From the findings, the development of the Saudi’s vision 2030 has incorporated the role of the healthcare sector development in attaining the healthcare goals (Abdelmaboud et al., 2022). Within the healthcare system, the focus would be on the investments made in the full realization of the healthcare quality outcomes. Vision 2030 has incorporated the National Transformation Program (NTP), which has been identified as an important aspect in incorporating the healthcare technologies in the management of healthcare processes. The focus has been on the creation of the procedures and strategies that would help working with technologies to improve healthcare outcomes (El-Sherif et al., 2022). The use of AI has therefore been developed through the priorities made to use technologies as part of improving the overall outcomes within the healthcare system. The current trends in the technologies have therefore been influenced by the opportunities environed in the healthcare deliverables under Saudi’s vision 2030.

### 4.2 What are the current challenges facing the use of artificial intelligence in Saudi Arabia’s healthcare system?

The challenges identified in this systematic review, including the lack of comprehensive AI-related policies, insufficient capital investment, and a cultural environment not fully integrating AI, were consistent with broader concerns regarding technology adoption within healthcare settings (Al-Jehani et al., 2021; Rong et al., 2020). These barriers impede the full realization of AI’s potential benefits, such as improved access to care, enhanced diagnostic accuracy, and more effective healthcare outcomes. Addressing these challenges requires a multi-faceted approach involving government, healthcare institutions, and technology providers (Alowais et al., 2023).

From the results, the development of the public healthcare system has focused on the accessibility to quality healthcare, which has minimized the realization of the intended goals in the healthcare technologies (Abdullah and Fakieh, 2020). The gaps in the full automation of the systems have been critical in establishing the challenges in benefiting from the use of AI in the healthcare system. One of the notable gaps is the need for investments in the AI systems, which is a challenge in the public healthcare system. Most of the facilities have been developing a gradual approach for managing and influencing the use of healthcare technologies. The results showed that the other gaps come in the cyber security issues, that have been a concern for the investors and users of the AI in Saudi Arabia (Aboalshamat et al., 2022). The government has posed tough guidelines for the information systems, which would affect the relative attractivity of AI, based on the perceived and anticipated security issues.

In the implementation of AI, the role of the healthcare workers, patients and investors remain critical. The skills gap among healthcare professionals and the inherent difficulties in adopting new technologies remain significant obstacles. As Amer et al. (2022a) emphasized, the active involvement of healthcare professionals in the evaluation process is critical for effective AI integration strategies. Therefore, targeted training programs, user-friendly AI interfaces, and ongoing support are crucial to bridging the skills gap and fostering a culture of AI acceptance. For the patients, lack of trust within the system and shift from the norms has been a challenge (Alotaibi and Alshehri, 2023). Lack of precise information and exposure to AI has therefore been an important barrier in ensuring that the patients can opt to use AI systems. For investors, the rapidity in the change in contemporary technologies leads to laxity in the investments into the AI in the system (Khafaji et al., 2022).

### 4.3 How is the future direction that would guide the use of artificial intelligence in Saudi Arabia’s healthcare system?

The results showed that the future of AI in Saudi’s healthcare system depends on the policies that would offers the incorporation of upcoming technologies into the healthcare system (Al-Jehani et al., 2021). However, while Al-Jehani et al. (2021) focus specifically on the Saudi context, similar calls for policy frameworks can be found in the World Health Organization, which has many policy documents about AI adoption (Chakraborty and Karhade, 2024). The political approaches come from the government’s considerations on the investments and the impacts. From the results, the development of the vision 2030 has been one of the strategic components for determining the future of AI in the country (Secinaro et al., 2021). The intention to use AI has been captured through the benefits that come with the quality of care and the efficiency. The development of the regulatory framework and the standards that would determine the incorporation of AI has been an important step towards the future of innovation (Chikhaoui et al., 2022). The development of the systems would be an effective way to ensure that the country influences technological use, while embracing contemporary technologies. The aim would be to have a strategic plan to ensure that the AI systems are regulated and can meet the population’s health needs (Chikhaoui et al., 2022).

Additionally, the results showed that the investments factor has always been a concern for the use of the various healthcare technologies. The investment would determine the chances and opportunities for having an efficient system for the integrated healthcare technologies (Nasseef et al., 2022). AI created a room for infrastructure development and research, which can help contextualize its use in the system. Saudi Arabia has been focusing on the use of the institutions of higher learning and training facilities to help build the required approaches for infrastructure development. The workforce development and training measures have been set through the inclusion of AI in the training approaches (Alowais et al., 2023). Therefore, to strengthen these advancements, future efforts must adopt a comprehensive approach that includes the development of well-defined policies and ethical guidelines (Al Momani et al., 2021; Abdullah and Fakieh, 2020), strategic investments in infrastructure, targeted skills development programs, and proactive public awareness initiatives (Amer et al., 2022b; Alowais et al., 2023; Saleem et al., 2023). Additionally, prioritizing research efforts and fostering robust collaboration through data-sharing frameworks are essential. Establishing national AI research centers dedicated to healthcare innovation will further drive progress in this field (Al-Jehani et al., 2021).

This systematic review highlights key lessons influencing AI implementation in Saudi Arabia’s healthcare system. The slow adoption of AI is significantly impacted by the absence of clear policies and governance frameworks, hindering responsible innovation and ethical considerations. To fully realize AI’s potential, strategic investments in infrastructure, data management, and workforce development are essential. Additionally, fostering cultural acceptance and addressing skill gaps through targeted education and training programs will enhance trust and promote widespread adoption among healthcare professionals and the public. Effective AI integration requires a focus on context-specific applications, such as clinical decision support systems, ensuring alignment with existing workflows and infrastructure.

## 5 Conclusion

This systematic review highlights that the integration of AI in Saudi Arabia’s healthcare system is at crucial development stage, influenced by several factors. The current utilization shows a gradual adoption pattern, primarily constrained by infrastructure investments and governmental prioritization of healthcare accessibility. Future research should prioritize several key areas to address identified challenges and advance AI implementation in healthcare. First, the development of clear ethical guidelines and regulatory frameworks is essential to ensure responsible and equitable use of AI in Saudi healthcare, particularly given the current policy gaps identified in the literature. Second, targeted training programs are needed to address the significant skills gaps among healthcare professionals, equipping them with the knowledge necessary to effectively utilize AI-powered tools. Third, research should explore AI applications in specific areas such as chronic disease management, telemedicine, and mental health, with emphasis on effectiveness and cost–benefit analyses. This systematic review also highlights the need for studies examining patient perspectives and trust-building mechanisms, as these factors significantly influence AI adoption rates.

The successful integration of AI in Saudi Arabia’s healthcare system is fundamental to achieving Saudi Vision 2030’s goals of technological advancement and knowledge-based economic transformation. Future research directions should include longitudinal studies tracking implementation outcomes, comparative analyses with regional healthcare systems, and investigations into cost-effective infrastructure development models.

## Data Availability

The original contributions presented in the study are included in the article/supplementary material, further inquiries can be directed to the corresponding author.
